# Communication at Transitions: One Audacious Bite at a Time

**DOI:** 10.2196/jopm.8924

**Published:** 2017-12-20

**Authors:** Daniel van Leeuwen

**Affiliations:** ^1^ Health Hats Arlington, MA United States

**Keywords:** Transitions, Quadruple Aim, care team, communication, leadership, e-patients, patient-physician relationship

## Abstract

To be audacious and take significant steps toward achieving the Quadruple Aim (improving the patient experience of care; improving the health of populations; reducing the per capita cost of health care; and improving the work life of clinicians and staff), we patients and caregivers need to better understand key features of our health journeys. When on that health journey, we are patients interacting with a series of care teams: our home team (social network), our community agency teams, our emergency care team, our hospital teams, and on and on. These care teams include ourselves, our caregivers, clinicians, other professionals, and direct care and support staff—people at the center of care. The actions taken by people at the center of care to improve, maintain, or adapt to our health or illness represents our health care. Actions can be diagnostic, taking medications, undergoing procedures, learning, living life and getting help living life. So, our health journey is teams of people at the center of care taking such actions to provide healthcare and service to us. During this journey, we transition from one setting to another, from one team to another, repeatedly. Communication knits this maze of actions, interactions, and transitions together. At its core communication is two or more people or parties sharing some information via some channel (voice, paper, digital, dramatic), one time or several times in a particular setting, hoping to accomplish something that moves us along in our health journey. One of the most persistent and ubiquitous frustrations in health care is that of poor communication. Poor communication at transitions is at the root of much overuse, underuse, and misuse of health resources, and results in the inability of patients to complete recommended treatment. For the patient and their family this means unnecessary delays in returning to health or worse. For those professionals on the care team the incidents of harm, burnout, stress, and frustration cause financial, emotional and career-ending consequences. Poor communication at transitions impacts each of the Quadruple Aims. The potential return for the investment in communication may cross over one or more organizational boundaries. Organization Boards and the C-Suite customarily focus on activities within their institutions, not between. The daunting nature of the challenge, caused by the shear volume and variety of transition nodes, can paralyze those in decision making roles, leading to smaller, more manageable local solutions. I support building a more holistic solution that includes the necessary governance, infrastructure, habits, and relationships. This leads to systematically applied common standards for local, node-specific solutions. Development should include all persons at the center of care in governance, design, operations and learning for systemic and local solutions. Refined clinical work flow should be constructed to respect patient and care partner life flow. Solutions should use interoperable technology to aid, not replace, communication. Transition information and processes should be transparent to patients and their care partners.

## The Big Picture

The Quadruple Aim [1], a unifying goal for the health care industry, is being widely adopted across the full continuum of care. The Quadruple Aim includes:

- Improving the patient experience of care;
- Improving the health of populations;
- Reducing the per capita cost of health care; and
- Improving the work life of clinicians and staff.

Some say that striving towards a goal that can never be totally reached is folly. I believe that working towards the Quadruple Aim represents an audaciousness needed to progress in solving our health care system’s challenges.

Facing such a large challenge, some are tempted to ignore the opportunity completely, some continue to do that which they already do well, and others nibble at the problem. This essay is for the people who believe in the inadequacy of these responses.

Anecdotes abound indicating that our current health care system has far to go before approaching success in achieving the Quadruple Aim. Statistics are readily available—but we won’t focus on them here; you may find or recall your own favorites! But here are two worth repeating:

- Approximately 30% to 50% of US adults are not adherent to long-term medications, leading to an estimated $100 billion in preventable costs annually [2].
- In 2011, there were approximately 3.3 million adult hospital readmissions in the United States associated with about $41.3 billion in hospital costs [3].

Where else inside of a modern organization can one witness such a wide range of people—from highly trained and specialized professionals to technical staff to housekeeping staff? Where else can you find such a high volume of patient (individual) interactions whose results can be life-or-death critical and may be time restricted to as little as 8 minutes? What industry is so complex that everyone finds part of it a complete mystery? What industry is projected to be the single largest segment of the US economy by 2024? Health care.

## Health as a Journey

To be audacious and take significant steps toward achieving the Quadruple Aim, we patients and caregivers need to better understand key features of our health journeys. When on that health journey, we are patients interacting with a series of care teams: our home team (social network), our primary care team, our specialist team(s), our community agency teams, our emergency care team, our hospital teams, and on and on. These care teams include ourselves, our caregivers, clinicians, other professionals, and direct care and support staff—people at the center of care. The actions taken by people at the center of care to improve, maintain, or adapt to our health or illness represents our health care. Actions can be diagnostic, taking medications, undergoing procedures, learning, living life, and getting help living life. So, our health journey is teams of people at the center of care taking such actions to provide health care and service to us. During this journey, we transition from one setting to another, from one team to another, repeatedly. The adult child of an elderly woman describes their journey:

I’m the child, custodian and health care proxy of my 89-year-old mother, Alice. I live in a different state. My mother has diabetes and is depressed. Her care team, beside herself and me, includes medical providers in various health settings, community support agencies, and a full-time caregiver who helps her schedule and get to health-related services. My problem is to understand what my mother wants for herself and to track who says they’re doing something for her (including my mother and me), what they’re doing, and when they’re doing it. I want to know what it takes to do it (Can she afford it? Can she get there? Does it agree with her? Who will be with her? etc). I want to know if the actions have the effects we thought they would. I want to know what her risks are and how we plan to prevent or respond to them. I want to able to keep track of all this and keep it current. I want to share it or have it shared from day to day and from setting to setting even if I’m not present.

## Communication at Transitions

Communication knits this maze of actions, interactions, and transitions together. At its core, communication is two or more people or parties sharing some information via some channel (voice, paper, digital, dramatic), one time or several times in a particular setting, hoping to accomplish something that moves us along in our health journey. One of the most persistent and ubiquitous frustrations in health care is that of poor communication.

These are some common complaints about communication, as expressed by real patients:

I don’t know when to call my doc or the hospital. What if I have new symptoms or questions about all these meds? Should I bother them on the weekend or at night?

I see six specialists in three different systems, all with portals, and I’m still the person who schleps information from one doctor to the other.

I was discharged after bypass surgery with 26 pages of instructions. I was just concerned about getting home.

Here, we are considering communication at transitions. Clinicians and patients, with their families, caregivers, and care partners, crave constant, collaborative, smooth, and sustainable communication during the health journey. Points of transition—where one person or group is being removed or added to the “team”—occur frequently and are the weakest link of communication in the care chain. I call these links “transition nodes.” These nodes include clinician hand-offs (day-to-day and shift-to-shift); communication from one clinician to another (eg, between nurse and doctor, primary care to specialist, pharmacist to nurse); transfers from one level of care to another (eg, hospital to home, community health to primary care); and care planning between clinicians and patients and their support networks (eg, discharge planning from acute care or into or from home to doctor office). Health care communication across transition nodes happens billions of times a day with great cumulative impact on lives, communities, well-being, and resources.

Poor communication at transitions is at the root of much overuse, underuse, and misuse of health resources, and results in the inability of patients to complete recommended treatment. For the patient and their family this means unnecessary delays in returning to health or worse. For those professionals on the care team the incidents of harm, burnout, stress, and frustration cause financial, emotional and career-ending consequences. Poor communication at transitions impacts each of the Quadruple Aims.

## What We Have Observed

The content, manner, and place of transition of care communication vary widely. The effectiveness of transition communication decreases as the difference between professions, departments, systems, and levels of care increases (nurse shift-to-shift strongest, across departments weaker, across systems and from acute or clinic to community based or home is weakest).

Communication at shift hand-offs between nurses can vary depending on age and experience of the nurses, their team dynamics, acuity and diagnoses of the patients. Thirty-year-old clinicians may communicate differently than 60-year-old clinicians. Intensive care unit (ICU) professionals share different information than emergency department (ED) professionals. And for all clinicians, there is a wide range in the degree of comfort about when to include patients and their families in discharge and care planning.

In general, a tension exists between the wealth of transition information needing to be communicated and the time needed to create, share, absorb, and understand that information. Acute care and clinic settings have the most time constraints. Chronic care and non-acute settings have more time, hence more opportunity for relationship building and person-centered information. Unfortunately, it seems that many organizations and teams only allocate adequate resources for improving transition communication after pain points have been reached or are threatened (harm, lawsuits, financial loss, public attention, and complaints) rather than proactively and systematically. Financial pressure to keep labor expense per patient as low as possible impacts effective communication.

## Current Efforts

Many tools and mnemonics exist to aid in the consistency of transition communication. Mnemonics, a memory device used to standardize and train many people during frequent encounters, are each designed for a specific setting/node of communication. A limited number have evidence as to their effectiveness [4]. Most contain identifying information, summary and current state, immediate plan, current or anticipated risk, and opportunity for learning (questions and synthesis).

Current published transition communication tools are predominantly acute care and medicine-centric [5]. Yet, transitions occur across the health continuum and with all members of the health team (licensed and non-licensed, professional and non-professional). Membership of a person’s health team can include the patient, their family and care partners, pharmacists, integrated health practitioners (chiropractors, massage therapists, nutritionists, etc), and community health agencies. Transitions increasingly occur outside of the hospital and traditional medical clinics, in settings such as mobile health, community and home, and retail walk-in and urgent care centers. In fact, such transitions increasingly include social services such as criminal justice, employment, housing, education, and child services. This is the extended continuum of care.

Organizations cited as exemplary in transition communication excel in one or maybe two nodes of transition. Technology could help, yet existing electronic health records are seldom interoperable or easily accessible at time of transition by all stakeholders. Hence, much communication still occurs on paper, via fax, by voice, or telephone. Too often the patient provides substantially all the communication and coordination.

## Solutions

Meeting the audacious goal of achieving the Quadruple Aim through collaborative, smooth, sustainable, and effective communication at all transition nodes in the health journey requires an infrastructure for implementing sustainable change to achieve success. This transition of care communication infrastructure includes patient and caregiver engagement, policies and standards, workforce management, technology, work flow and life flow, governance, and learning (see Textbox 1).

Sustainable implementation of transition communication can be tool agnostic. Building the infrastructure is an iterative, growing, learning endeavor with common system and leadership requirements. Designing the work flows and tools for specific transition nodes has unique local, operational components depending on the participants, the setting, and the culture.

Standards do exist for building and sustaining effective transition communication and communication tools. One example is the Joint Commission’s SHARE Solutions that provides an approach for developing and evaluating hand-off tools (see Textbox 2) [6]. In addition, care planning has generally accepted components (see Textbox 3).

Establish standards for transition plans—create each plan with all parties involved including family and personal care partners and destination facilities. Document those plans with communication channels suitable to the users, lay and professional. Such standards could address a wide range of communication barriers, from external barriers like distinct electronic systems to internal barriers like age-related communication issues.

Some hospitals and clinics implement interactive voice response (IVR) calls and multimedia programs for transition communication.

Finally, the only consistency across transition nodes is the patient and their family and personal care partners, yet the industry is only beginning to include them in communication planning, work flow, learning, and technology. The Institute of Medicine (IOM) recommends that patients become a full partner in their own care. Patients can be an important safety net in catching errors before they lead to harm.

**Transition communication infrastructure.**

**Patient and Caregiver Engagement:** Patients and caregivers participating in design, operations, governance, and learning.

**Policies and Standards:** Play-book or standard approach for the key elements of information, data, tracking, and work flow regarding communication in each node setting.

**Workforce:** All people at the center of care and their leaders hired for, aligned, and committed to collaborative, smooth, sustainable transition communication every time.

**Technology:** All electronic communication vehicles and channels (such as, EHR, portals, web sites, messaging, phones, faxes) synchronized and interoperable to support transition communication and care planning.

**Work flow:** Clinician, direct care, and support staff work processes proactively designed for efficient and effective communication at all nodes.

**Life flow:** Clinicians, direct care and support staff appreciate the complexity and context of patient and caregiver lives as they manage health away from professionals.

**Governance:** Explicit accountabilities for transition communication at each node and overall with resources allocated to support.

**Learning:** Standardized orientation and continuing education of all transition dyads within and across professions, departments, and organizations. Routine measurement and analysis of transition communication effectiveness with sharing of lessons learned to all stakeholders.

**The Joint Commission Center for Transforming Healthcare’s SHARE Solutions [ 6].**

1. **S**tandardize critical content.
2. **H**ardwire within your system.
3. **A**llow opportunity to ask questions.
4. **R**einforce quality and measurement.
5. **E**ducate and coach.

**Care planning components.**

- What needs to happen?
- By whom, by when?
- Goals/Expected outcomes?
- Anticipated risks and amelioration strategies
- Barriers

Communication usually occurs in dyads—a dynamic of two individuals or two teams (nurse-nurse, patient-doctor, hospital-nursing home, etc). Each individual or team in the dyad can have widely varied comfort and skill in that communication. That variation occurs for clinicians, support staff, patients, families, and site of care. Individuals and teams need to take their dyad partner where they are and persistently increase comfort and skill. This means first, understand the stages of skill and comfort (engagement, activation, background, experience), next quickly assess your dyad partner’s stage, and then fine-tune the communication to that assessment. All this requires learning and continuous improvement: orientation, training, continuing education, coaching, process and outcome measurement, and work flow refinement.

## Final Thoughts

The Quadruple Aim can be significantly accelerated by effective communication at transitions. Why don’t health care organizations invest more in comprehensive, sustainable solutions? I believe the potential return for the investment in communication may cross over one or more organizational boundaries. Organization boards and the C-suite customarily focus on activities within their institutions, not between. The daunting nature of the challenge, caused by the shear volume and variety of transition nodes, can paralyze those in decision making roles, leading to smaller, more manageable local solutions.

I support building a more holistic solution that includes the necessary governance, infrastructure, habits, and relationships. This leads to systematically applied common standards for local, node-specific solutions. Development should include all persons at the center of care in governance, design, operations and learning for systemic and local solutions. Refined clinical work flow should be constructed to respect patient and care partner life flow. Solutions should use interoperable technology to aid, not replace, communication. Transition information and processes should be transparent to patients and their care partners.

Critical to success:

1. Board and C-suite *prioritize*
2. All levels of management *accountable*
3. People at the center of care *included* at every step
4. Persistently and continually *improve and apply lessons learned*
5. Emphasize *transparency of information and processes* to all stakeholders.

As a patient or caregiver, you can make a difference.

1. When you are well enough and have space in your life, *get a seat at the table*. Pick a table that suits you. You may have had an unsettling or delightful experience at a hospital, clinic, agency—any setting. You may be good at governance, design, publicity—you know what you are good at. Speak with the boss—the Executive Director, CEO, chief physician. Ask to join the board, the Patient Advisory Committee, wherever decisions are made.
2. Pay attention to communication at transitions. Ask to shadow people at the center of care. Ask questions. See where communication works and where it doesn’t. Put the topic on the agenda and share what you’ve learned.
3. When you are a caregiver of a patient in a hospital or going to the doctor or moving along a health journey, pay close attention to communication at transitions. Who is communicating with whom? How is the patient involved; how are you involved? Ask questions. Find out who to contact when you leave that setting. Expect a name, number, or link. Questions always come up even if you record everything.

You’re on your way! It’s worth it for patient experience, clinician well-being, population health, and every bottom line.

## References

[1] Sikka R, Morath JM, Leape L (2015). The Quadruple Aim: care, health, cost and meaning in work. BMJ Qual Saf.

[2] Marcum ZA, Sevick MA, Handler SM (2013). Medication nonadherence: a diagnosable and treatable medical condition. JAMA.

[3] Hines AL, Barrett ML, Jiang HJ, Steiner CA (2014). HCUP Statistical Brief #172.

[4] Li P, Ali S, Tang C, Ghali WA, Stelfox HT (2013). Review of computerized physician handoff tools for improving the quality of patient care. J Hosp Med.

[5] Levine C, Feinberg L (2013). Generations.

[6] Joint Commission Center for Transforming Healthcare (2012). Joint Commission Center for Transforming Healthcare releases targeted solutions tool for hand-off communications. Jt Comm Perspect.

